# Cancer Initiation, Progression and Resistance: Are Phytocannabinoids from *Cannabis sativa* L. Promising Compounds?

**DOI:** 10.3390/molecules26092668

**Published:** 2021-05-02

**Authors:** Ersilia Nigro, Marialuisa Formato, Giuseppina Crescente, Aurora Daniele

**Affiliations:** 1 Dipartimento di Scienze e Tecnologie Ambientali, Biologiche e Farmaceutiche, Università della Campania “Luigi Vanvitelli”, Via G. Vivaldi 42, 81100 Caserta, Italy; nigro@ceinge.unina.it (E.N.); marialuisa.formato@unicampania.it (M.F.); giuseppina.crescente@unicampania.it (G.C.); 2 CEINGE-Biotecnologie Avanzate, Via G. Salvatore 486, 80145 Napoli, Italy

**Keywords:** *Cannabis sativa* L., phytocannabinoids, inflammation, cancer therapeutic agents

## Abstract

*Cannabis sativa* L. is a source of over 150 active compounds known as phytocannabinoids that are receiving renewed interest due to their diverse pharmacologic activities. Indeed, phytocannabinoids mimic the endogenous bioactive endocannabinoids effects through activation of CB1 and CB2 receptors widely described in the central nervous system and peripheral tissues. All phytocannabinoids have been studied for their protective actions towards different biological mechanisms, including inflammation, immune response, oxidative stress that, altogether, result in an inhibitory activity against the carcinogenesis. The role of the endocannabinoid system is not yet completely clear in cancer, but several studies indicate that cannabinoid receptors and endogenous ligands are overexpressed in different tumor tissues. Recently, in vitro and in vivo evidence support the effectiveness of phytocannabinoids against various cancer types, in terms of proliferation, metastasis, and angiogenesis, actions partially due to their ability to regulate signaling pathways critical for cell growth and survival. The aim of this review was to report the current knowledge about the action of phytocannabinoids from *Cannabis sativa* L. against cancer initiation and progression with a specific regard to brain, breast, colorectal, and lung cancer as well as their possible use in the therapies. We will also report the known molecular mechanisms responsible for such positive effects. Finally, we will describe the actual therapeutic options for *Cannabis sativa* L. and the ongoing clinical trials.

## 1. Introduction

*Cannabis sativa* L. (Hemp) is a plant long used for its textile fibers and seed oil. Beyond these uses, it is the main source of over 150 active compounds known as phytocannabinoids [1] which have received renewed interest in recent years due to the diverse pharmacologic activities such as anti-inflammatory effects, cell growth inhibition, and tumor regression. Among the others and although not properly constitutive in hemp, the most representative compounds are Δ^9^-tetrahydrocannabinol (THC) and cannabidiol (CBD). The former, THC appears the most active [2,3] although its use is limited by the psychotropic effects it exerts, whereas CBD is the most abundant neutral form among phytocannabinoids in hemp. Compared to THC, CBD has non-psychoactive effects, an advantage for clinical applications. CBD has become extraordinarily popular around the world, being commercially available as dietary supplements, creams, lotions, and the most commonly used oils [4,5]. Furthermore, acidic cannabinoids such as cannabidiolic acid (CBDA) and tetrahydrocannabinol acid (THCA), together with cannabigerolic acid, are the main phytocannabinoids in hemp [6,7]; they lack psychoactive effects, and undergo decarboxylation by heat or aging. Another phytocannabinoid, less expressed in *Cannabis sativa* L., is cannabinol (CBN) which is a degradation product of the *Cannabis* metabolite Δ^9^-tetrahydrocannabinol, with a concentration in cannabis between 0.1 and 1.6% [8]. It is quite important to notice that a synergistic interaction of cannabinoids with terpenes and flavonoids has been proven. Indeed, terpenes are known to modulate THC pharmacokinetics by increasing blood–brain barrier (BBB) permeability [9]. Ratios between terpenoids and phytocannabinoids may substantially improve potential medical therapies [10]. Secondary metabolites may affect THC affinity for the CB1 receptor; flavonoids may also potentially affect THC pharmacokinetics [11].

Phytocannabinoids exert their biological effects by mimicking the actions of a family of endogenous bioactive mediators named endocannabinoids that activate two specific G protein–coupled cannabinoid receptors: CB1 and CB2 [12,13]. Both expression and function of CB1 have been widely described in the central and peripheral nervous system but their expression is present also in other peripheral tissues [14,15]. CB1 is a Gi/o type of GPCR that inhibits adenylyl cyclase (AC) activity and chunks the pathway of cyclic adenosine monophosphate (cAMP) and protein kinase A (PKA). Furthermore, CB1 suppresses the influx of Ca^2+^ ions and activates several components of the mitogen-activated protein kinases (MAPK) family and phosphoinositide-3-kinase/protein kinase B (PI3K/AKT) pathway [14,16]. CB2 is a GPCR-associated receptor, expressed as two isoforms, A and B. The isoform CB2A is mainly found in the testis and lower brain regions, while CB2B is more present in tissues of the immune system [13]. In addition, THC is a partial agonist of CB1 and CB2 receptors while CBD has greater affinity for CB2 than CB1 [17,18].

Nowadays, cannabinoids are being investigated as potential therapeutic agents for different pathologies [19], including cancer [20]. Indeed, both receptors, CB1 as well as CB2, are expressed in several cancer types including lung, breast and prostate cancer, glioblastoma, and colorectal cancer, further demonstrating an implication of the endocannabinoids system in cancer [16,21,22,23]. Furthermore, although CBN, CBG, and THCA effects have been less explored so far, all phytocannabinoids show protective effects towards a number of biological mechanisms, including inflammation, immune response, and oxidative stress. Altogether these effects result in an inhibitory activity against cancer [20]; on the other hand, emerging evidence demonstrated that phytocannabinoids can also modulate tumor growth through regulation of biological responses strictly related to the carcinogenesis process, such as inflammation, oxidative stress, and apoptosis [24].

In this scenario, the aim of this review was to report the current knowledge about the action of phytocannabinoids in *Cannabis sativa* L. against cancer initiation and progression with a specific regard to brain, breast, colorectal, and lung cancer, as well as their possible use in the therapies, substantially reducing the adverse effects. Furthermore, we reported the known molecular mechanisms responsible for such beneficial effects. Finally, we described the actual therapeutic options for *Cannabis sativa* L.

## 2. The Chemistry of Hemp

**Figure 1:**
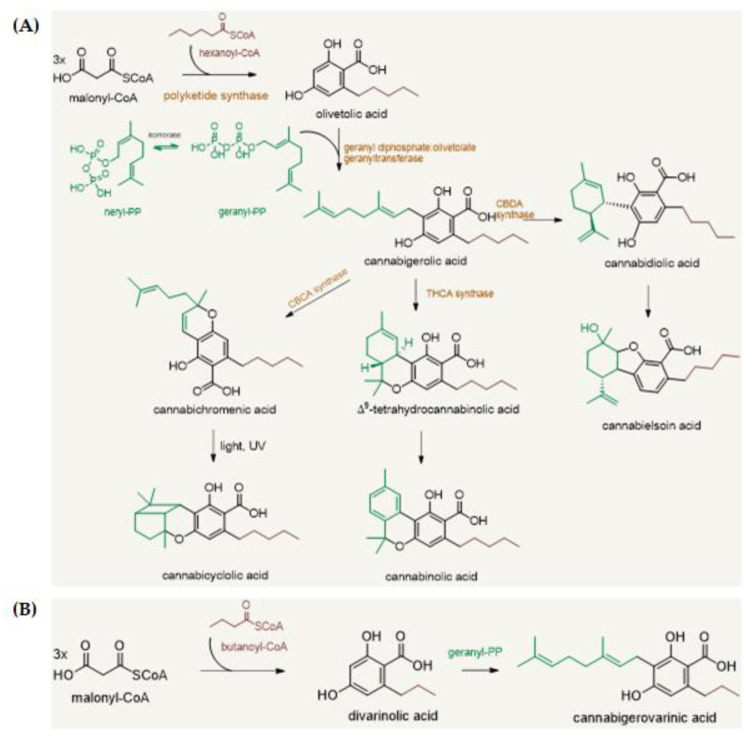
(**A**) General overview of cannabinoids’ biosynthetic pathway; (**B**) Butanoyl-CoA is involved in the biosynthesis of propyl cannabinoids, of which cannabigerovarinic acid (CBGVA) is the precursor compound.

**Figure 2:**
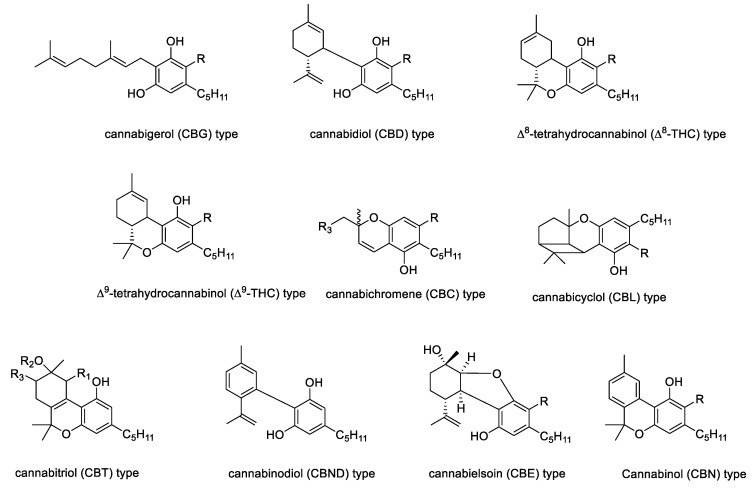
Cannabinoid type derived from an olivetoid backbone. R = H (neutral cannabinoids); R = COOH (acidic cannabinoids).

The phytochemistry of industrial *Cannabis sativa* L. is very complex with more than 500 secondary metabolites isolated and identified, of which a considerable number belongs to the phytocannabinoid class [25]. Phytocannabinoids are terpenophenolic compounds, whose carbon structure is mainly constituted by 22 or 21 carbon atoms, likely an alkyl resorcinol linking a monoterpene moiety (Figure 1). The polyketide pathway leads to an alkylresorcinol following the reaction between hexanoyl-CoA with three molecules of malonyl-CoA to yield olivetolic acid (OA). This latter undergoes prenylation by geranyl diphosphate (GPP), that derive from the deoxyxylulose phosphate/methylerythritol phosphate (DOXP/MEP) pathway [26]. Thus, through the cannabigerolic acid synthase (CBGAS) enzyme, mainly expressed in the glandular trichomes of female flowers, cannabigerolic acid (CBGA) is biosynthetized; this last is considered the precursor of all the phytocannabinoids [6,26]. Subsequently, CBGA is converted into CBDA, CBCA, and Δ^9^-THCA, through the action of flavinylated oxidases, namely cannabidiolic acid synthase (CBDAS), cannabichromenic acid synthase (CBCAS), and Δ^9^-tetrahydrocannabinolic acid synthase (Δ^9^-THCAS) [27]. Indeed, the versatility of phytocannabinoid biosynthesis augments the compounds’ structure variability, which further is affected by UV-light and temperature. Thus, phytocannabinoids differ into ten different main types (e.g., CBD-, Δ^9^/Δ^8^-THC-, CBG-, CBN-, CBND, CBT-, CBL-, CBE-, or CBC-type (Figure 2). A “miscellaneous” type is also reported, to which some different molecules, not chemically complying with the most common phytocannabinoids, such as cannabifuran (CBF-C5), dehydrocannabifuran (DCBF-C5), cannabicoumaronone-C5 (CBCON-C5).

Acetyl-CoA and butanoyl-CoA also react with malonyl-CoA derived-polyketide to produce other cannabinoid derivatives. In this context, a well-known example is cannabidivarinic acid, which could be considered a CBDA propyl analogue [7]. Moreover, acidic phytocannabinoids could undergo nonenzymatic decarboxylation to yield the neutral forms, which often retain or enhance the bioactivity of their precursors.

Beyond phytocannabinoids, *Cannabis sativa* L. is a source of a plethora of other compounds with pharmacological potential [28] such as terpenoids, alkaloids, and polyphenols (e.g., flavonoids, stilbenes, phenylpropanoid amides, lignanamides) [29]. In hemp, different flavonoids, that have been identified from pollen, leaves, and flowers, have as aglycone quercetin, kaempferol, orientin, apigenin, and luteolin [30]; these flavonoids could be present as *C*- or *O*-glycoside conjugates. Methylated prenylated flavones, cannflavins A, B, and C, as well as flavonols glycosides, such as kaempferol 3-*O*-sophoroside and quercetin 3-*O*-sophoroside, were also isolated from hemp pollen [31]. Furthermore, from *Cannabis sativa* L. fruits, beyond to a source to produce an oil rich in tocopherols and mono-, di-, and polyunsaturated fatty acids (PUFA) such as α-linolenic (ALA), γ-linolenic acid (GLA), and stearidonic acid (SDA), contained different phenylamides, tyramine and octopamine derivatives of hydroxycinnamic acids, and their lignanamides, also called cannabisins [32,33].

## 3. A Brief Focus on Endocannabinoid System

**Figure 3:**
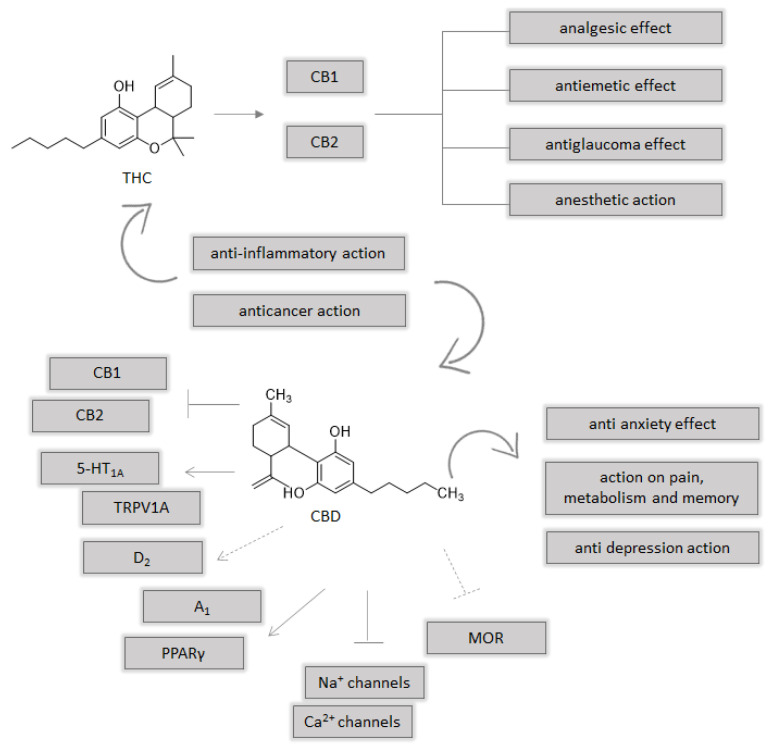
Multiple molecular targets for THC and CBD; agonist →; antagonist ꟷꟾ; negative allosteric modulator ---⁞; inhibitor ---ꟾ; partial agonist --->.

The endocannabinoid system (ES) plays a key role in the inflammatory processes, and includes CB1 and CB2 cannabinoid receptors. The first endogenous cannabinoid ligands (eCBs), intensively investigated, are arachidonoylethanolamine (anandamide or AEA) and 2-arachidonoylglycerol (2-AG) [21]. Furthermore, ES comprises different enzymes involved in the synthesis, reuptake, and degradation of cannabinoids. Anandamide is synthesized from *N*-acyl-phosphatidylethanolamine (NAPE) by the enzyme NAPE-specific phospholipase D (NAPE-PLD), while 2-AG is synthesized from diacylglycerol (DAG) by DAG lipase (DAGL). The endocannabinoid system could be a target for different pathologies, in particular for cancer, with different levels of dysregulation that can involve cannabinoid receptors or the enzymes [34]. The most important enzyme involved in eCBs degradation are fatty acid amide hydrolase (FAAH) for anandamide and monoacylglycerol lipase (MAGL) for 2-arachidonoylglycerol. Beyond CB1 and CB2 cannabinoid receptors, other targets are suggested, such as GPR55 [35], transient receptor potential vanilloid 1 (TRPV1) ion channel [36], and peroxisome proliferator-activated receptor (PPAR) α and γ localized in the nucleus [37]. The CB1 and CB2 receptors are coupled to G protein, whose activation leads to an inhibition of adenyl cyclase, decreased production of cAMP, and variation of ion channel activity. THC is the most abundant cannabinoid and the first psychoactive constituent that was isolated in 1964 [38], which binds CB1 and CB2 receptors. THC, with its metabolite THC 11-oic, could explain analgesic, antiemetic, and antiglaucoma effects or anesthetic action [39]. Unlike THC, CBD acts as an antagonist of CB1 and CB2 receptors and its activity on the endocannabinoid system could be explained by its inhibition of FAAH enzyme for increase of endocannabinoids, such as anandamide, that normally have a short shell life. Beyond the action on the endocannabinoid system, there are different targets of CBD such as 5-HT1A, TRPV1A, D2, A1, MOR, PPAR γ, sodium, and calcium channels [40]. These different endogenous targets underline the CBD actions on anxiety, depression, pain, memory, and metabolism (Figure 3). The activation of CB1/2 receptors and transient receptor potential (TRP), maybe vanilloid 1, could inhibit some cancer cell invasion and metastasis, acting on different pathways involved in the angiogenesis, tumor vascularization, and tumor cells ability to destroy matrix membranes. Nuclear receptors PPAR, in particular PPARα and PPARγ, and the receptor GPR55 can be considered targets of cannabinoids also in cancer. Moreover, cyclooxygenase 2 (COX-2) can play a critical role on the behavior of endocannabinoids on cancer [41].

## 4. Molecular Effects of *Cannabis sativa* L.

Several molecular processes are strictly related to cancer initiation and development including inflammation, oxidative stress, and proliferation. Cannabinoids exert a number of beneficial pharmacological effects, including anti-inflammatory and antioxidant properties [42].

Several studies indicate that cannabinoid receptors and endogenous ligands are overexpressed in tumor tissues [43]. Moreover, increased expression of enzymes involved in endocannabinoid metabolism is often associated with the aggressiveness of cancer. [44,45]. Cannabinoids target the tumor affecting signaling and cellular pathways such as tumor cell proliferation, angiogenesis, tumor invasion, and apoptosis both in in vitro and in vivo experiments [46]. In particular, different evidence suggests that these compounds exert inhibition of initiation, progression, and metastatic capacity of several cancer types [17,43,47,48].

Both THC and non-psychoactive cannabinoids have been reported to possess peripheral anti-inflammatory properties in a plethora of in vitro and in vivo models [49,50,51]. In human peripheral blood cells, CB1 is expressed by B cells, NK cells, neutrophils, CD8^+^ T cells, monocytes, and CD4+ T cells, whereas CB2 mRNA is expressed by human B cells, NK cells, monocytes, neutrophils, and T cells [52]. Typically, CB2 inhibits the production of proinflammatory cytokines, such as tumor necrosis factor alpha (TNF-𝛼), interleukin (IL)-2, IL-6, IL-8, and IFN-𝛾 by immune cells [51]. CBD decreases peripheral inflammation through reduction of prostaglandin E2 (PGE2), nitric oxide (NO), and malondialdehyde production [53,54,55,56,57]. In addition, CBD, in combination with minor phytocannabinoids of *Cannabis sativa* L. extracts, can induce a greater pharmacological anti-inflammatory activity [52,58]. Indeed, a standardized cannabis extract enriched with CBD exerts a more powerful anti-inflammatory activity than CBD alone [59]. Besides CBD, THC also possesses potent anti-inflammatory properties both in vivo and in vitro [60,61]. Recently, in a mouse model of acute respiratory distress syndrome, THC leads to the suppression of the cytokine storm [62]. The molecular mechanisms at the basis of THC down-regulation of the inflammatory processes are various and tissue-dependent [61]. Indeed, regarding gastrointestinal and systemic inflammatory reactions, THC suppresses both lymphocytes and neutrophils activity [63,64]; in epithelial and skin cells, THC inhibits the release of inflammatory mediators through impairment of the nuclear factor kappa-light-chain-enhancer of activated B cells (NF-kB) pathway [65]. It is of note that there is clear evidence of the synergistic action of THC and CBD in terms of down-regulation of the inflammatory processes [66,67].

Regarding other combination extracts, Shebabya et al. demonstrated that *Cannabis sativa* L. oil extract markedly suppresses the release of TNF-α in LPS-stimulated rat monocytes with inhibition of LPS-induced COX-2 and i-NOS protein expression and blockage of MAPKs phosphorylation [68]. Additionally, the presence of phenols, terpenes, or other phytocannabinoids enhance the therapeutic activity of CBD, defined as ‘entourage effects’ [69,70,71]. In addition, cannabis extract inhibits the production of IL-8, matrix metallopeptidase (MMP)-9, and vascular endothelial growth factor (VEGF), an effect not detected with CBD alone, in skin cells [65]. Other non-psychoactive cannabinoids, including CBC and CBN, also showed substantial in vivo anti-inflammatory responses. On the other hand, monoterpenes such as α- and β-pinene, myrcene, and limonene have been also reported to possess substantial anti-inflammatory properties [72,73,74].

Regarding neuroinflammation, both CBD and THC have protective effects [75,76,77] through the activation of NF-𝜅B as well as the inhibition of Toll like receptor (TLR4) [78,79]. Indeed, in a vitro model of LPS-stimulated neuroinflammation, CBD suppresses the release of TNF-α, IL-1β, and IL-6 through the inhibition of NF-𝜅B phosphorylation and the concomitant activation of COX and iNOS [57,80]. In addition, THC treatment selectively reduces CD8^+^ T cell response accompanied by inhibition of IL-6 release [81]. The combination of THC and CBD seems to be the most potent anti-inflammatory drug able to inhibit the T helper response as well as CD4^+^ T response in a mouse model of multiple sclerosis (MS) [82].

Beyond the regulation of inflammation, phytocannabinoids can prevent proliferation, metastasis, and angiogenesis, as well as induce apoptosis in a variety of cancer cell types [83,84,85]. Treatments with CBC and THC or CBD led to cell cycle arrest and cell apoptosis. Additionally, CBC and THC or CBD treatments inhibit bladder urothelial carcinoma cell migration and affected F-actin integrity [86].

Beyond the actions of CBC, THC, and CBD on different pathways involved into development of cancer cell types, also cannabigerol (CBG), cannabidivarin (CBDV), cannabinol (CBN), cannabivarin (CBV), and tetrahydrocannabivarin (THCV) have showed a role as anti-cancer for different cells line [34].

Besides the anticancer effects, a role in the resistance to chemotherapy has also been suggested for *Cannabis sativa* L. [87]. P-gp is exclusively over expressed in cancer cells leading to multidrug resistance (MDR) [88]. Phytocompounds of *Cannabis sativa* L. can influence Pgp activity. Indeed, in multidrug resistant mouse lymphoma cells, cannabinol, cannabispirol, and cannabidiol increase cytotoxic drug accumulation [89]. Furthermore, Kazemi et al. showed that the lignanamides cannabisin M and cannabisin N have high binding affinities to Pgp, suggesting an inhibitory effect toward MDR [90].

From a molecular point of view, phytocannabinoids mainly stimulate molecular targets deeply involved in tumor development and progression such as the G-protein coupled receptors, peroxisome proliferator-activated receptors (PPARs), glycine receptors (GlyR), and transient receptor potential channels (TRP) channels [36].

In the next paragraphs, we will deepen the current knowledge about the in vitro effects of phytocannabinoids on different cancer cells with a particular regard to CBD and THC and the molecular mechanisms that underlie the inhibitory actions.

### 4.1. Brain Cancer

Both CBD and THC are promising compounds in the fight against brain cancers. Deng et al. showed that CBD induces a dose-dependent reduction of both proliferation and viability on glioblastoma multiform (GBM) cancer cells, with a IC50 ranging between 3.1 and 8.5 µM. In addition, co-treatment of CBD with DNA damaging agents produces synergistic anti-proliferating and cell-killing responses in GMB cell line [91]. These data have been confirmed in the U-87 glioblastoma cell line in which CBD led to a concentration-related inhibition of the U87 cell viability [92]. In accordance, Nigro et al. analyzed, in the same glioblastoma cell line, the effects of a heterogenous extract from *Cannabis Sativa* L., finding the inhibition of both proliferation and migration from a dose of 25 µg/mL [32]. The underlying molecular mechanism is not completely clear but the authors evidenced DNA damage [32]. Recently, Singer et al. demonstrated that apoptosis together with ROS production are two additional mechanisms involved in CBD inhibitory activity of 3832 and 387 glioma primary stem cell lines (GSC) with an IC50 value of 3.5 μM [93]. The induction of apoptosis has also been confirmed by Alharris et al. in neuroblastoma SH-SY5Y and IMR-32 cell lines in which a reduction of cancer cell migration and invasion was also induced already with a dose of 10 μM [94]. A recent study suggested a role for CBD in interfering with chemoresistance in glioblastoma cells describing a decrease in prohibitin (PHB) and extracellular vescicles (EVs). EVs are lipid bilayer-enclosed structures which participate in cell-to-cell communication, both in physiological and pathophysiological processes regulating cell migration, differentiation, and angiogenesis and therefore playing an important role in cancers. Kosgodage et al. show that CBD reduces PHB protein levels and changes EV-mediated export of microRNAs to an anti-oncogenic signature in GBM cells [95].

Interestingly, it is to notice that cannabinoid treatment cannot affect cell viability of astrocytes (normal glial cells) in comparison to GBM cells, demonstrating a selectivity towards cancer cells. However, the molecular mechanisms mediating cannabinoid selectivity are not yet fully understood [96,97].

Regarding THC, similar anti-cancer effects have been demonstrated: Blázquez et al. found that, in mice and glioma cells, THC inhibits the growth and invasion of gliomas through the down-regulation of matrix metalloproteinase (MMP-2 expression), factor involved in the acquisition of invasiveness [98]. The anti-cancer activity of THC on recurrent GBM has also been demonstrated in vivo [99]. The mechanism underlying the THC anticancer properties has been only partially clarified and relies on the stimulation of an ER stress-related signaling pathways that unleash the autophagy-mediated cancer cell death [20,100]. Whether CBD or THC are more potent in antineoplastic activity on brain cells is still a matter of debate. Marcu et al. conducted experiments comparing the two molecules, concluding that CBD is a more potent inhibitor than THC in different glioblastoma cell lines (U87-MG, U251, and SF126) [101].

Besides the use of single cannabinoid, Baram et al. described cannabis extracts as antitumor agents in U-87 MG and T98G glioblastoma cell lines able to impair the survival and proliferation of cancer cells as well as induced apoptosis, to a greater extent than THC alone [102]. In addition, in a combination study, Valero et al. reported that a CBD concentration higher than THC (5:1), in combination with temozolomide [CBD (15 mg/kg) and TMZ (5 mg/kg)], targeted glioma stem cells in vivo much more efficiently than the THC/CBD formulation [103]. Remaining on the evaluation of combinational treatments, Ivanov et al. demonstrated the upregulation of the cytotoxic effect of γ-irradiation in GBM by the co-treatment with CBD. The dose of CBD treatment ranged between 5 and 20 µM, in accordance with previous results. The authors also found that CBD treatment substantially upregulated TNF/TNFR1 and TRAIL/TRAIL-R2 signaling by modulation of both ligand and receptor levels followed by apoptosis. The pathways triggered by CBD are JNK1/2 and MAPK p38 levels with the subsequent downregulation of the active phospho-ERK1/2 and phospho-AKT levels [104,105]. On the contrary, in a different cellular model, the U251 cell line, Marcu et al. showed that CBD did not increase the activity of JNK1/2 or p38 MAPK [101].

In addition to irradiation, CBD has also been tested with alkylating agents, especially TMZ, proving that together they have synergistic anti-proliferative effects on glioma cells [52,60,61,65].

Beyond proliferation and apoptosis, several papers reported in the GBM cell line an induction of oxidative stress by CBD treatment, accompanied by a decrease of the antioxidant cell potential, [106,107]. Interestingly, there is no ROS increase in CBD treated normal glial cells [107].

### 4.2. Lung Cancer

Cannabinoids and their agonists have been proposed as complementary pharmacological agents in the treatment of lung cancer thanks to their antineoplastic, apoptotic, and anti-metastatic properties [43]. A recent work by Milian et al., showed that lung tumors can be classified according to the expression of CB1 and CB2 receptors because patients with high expression levels of both receptors are associated with a better prognosis of the disease and survival [108].

In vitro studies have largely evidenced that both CBD and THC inhibit viability as well as invasiveness of lung cancer cells [109,110]. Ramer et al. demonstrated that CBD caused a profound inhibition of viability and invasion capacity of A549 and H460 lung cancer cells, accompanied by a decreased expression and secretion of PAI-1, at very low concentrations (as low as 0.1 μM); these events are CB1-, CB2-, and TRPV1-dependent. The authors also found that CBD reduces in vivo the size of tumor in nude mice [109].

Similar to CBD, THC has been found to suppresses viability and invasiveness of three different lung cancer cell lines (H1299, A549, H1975) deterring cell migration and abolishing cytoskeleton reorganization/focal adhesion assembly at a dose of 10 μM [111]. The molecular mechanism of action of THC passed through the abolishment of Src-dependent cytoskeleton reorganization and focal adhesion assembly diminished, both processes deeply involved in carcinogenesis and metastasis [24].

Recently, *Cannabis Sativa* female flower heterogenous extracts have been found to induce death of lung cancer A549 cell line in a time-dependent manner but at very low doses (50–900 ng/mL), following induction of early apoptosis, cell cycle arrest, elevation of ROS level, and activation of caspase 3 [112]. The mechanism seems to be mediated by the binding to the CB2 receptors, since their blockage caused attenuation of *Cannabis Sativa* effects on A549 cells [112].

Similar to what was described in brain cancer cells, besides the use of single cannabinoids, Baram et al. described cannabis extracts as antitumor agents in A549 and NCI-H460 lung carcinoma to a greater extent than THC alone [102]. The underlying molecular mechanisms and signaling pathways are mainly related to the activation of the extracellular signal-related kinase (ERK), phosphoinositide3-kinase (PI3K), p38mitogen-activated protein kinase (p38MAPK), and ceramide pathways [113]. Another mechanism demonstrated in CBD-treated lung cancer is the up-regulation of cyclooxygenase-2 (COX-2) and PPAR-gamma in vitro as well as in vivo [110]. Regarding the anti-metastatic activity, two mechanisms, directly correlated with the invasion process, have been proposed to be induced by cannabinoids: a) decreased secretion of plasminogen activator inhibitor-1 (PAI-1) [109]; b) an upregulation in the expression of ICAM-1 [114].

### 4.3. Breast Cancer

In preclinical studies, CB1 and CB2 agonists (CBD, THC, and synthetic) have been shown to inhibit the proliferation of estrogen receptors positive breast cancer cell lines [14]. Accordingly, a cytotoxic effect of CBD was observed in several cell lines including estrogen-receptor (ER)-positive cells (MCF-7, ZR-75-1, T47D), ER-negative cells (MDA-MB-231, MDA-MB-468, and SK-BR3), and triple-negative breast cancer cells (SUM159, 4T1up, MVT-1, and SCP2) [115,116,117,118,119].

In particular, CBD induces apoptosis and reticulum stress in MDA-MB-231 and MCF7 cancer cells inhibiting their growth [119]. Other studies confirmed these data in several breast cancer cell lines, showing inhibition of cell growth, DNA fragmentation, and apoptosis [120,121]. In addition, CBD treatment induces an enhancement in the level of ROS in breast cancer cells [70]. Furthermore, CBD induces apoptosis and blocks cell viability through the inhibition of the AKT/mTOR axis and cyclin D together with the enhancement of ROS generation [115,116]. Similarly, THC showed anti-proliferative effects on several breast cancer cell lines [116,122]. Elbaz et al. observed that CBD specifically inhibits the epidermal growth factor-induced proliferation, suggesting it as a novel potential therapeutic option for breast cancer [115].

Interestingly, CBD has been associated to sensitivity to chemotherapy; in MDA-MB-231 cells, it significantly reduces exosome release and inhibits microvesicle release [123] at a concentration of 1 and 5 μM. These regulatory processes may be associated with changes in mitochondrial function, including modulation of STAT3 and prohibitin expression, candidating CBD as a molecule useful to sensitize breast cancer cells to chemotherapy.

Besides natural compounds, synthetic agonists for cannabinoid receptors (WIN55, 212-2, and JWH-133) have also been tested in breast cancer showing dose-dependent anti-proliferative and anti-migration effects [123,124,125,126]. Comparably to natural ones, the effects induced by the agonists might be linked to the induction of autophagy and inhibition of cell-cycle progression through the enhancement of ROS production [119,126].

Regarding the minor phytocannabinoids, in MDA-MB-231 breast carcinoma cells, both CBG and CBC were shown to inhibit cell growth, inhibiting the uptake of [14^C^]anandamide and activating the vanilloid receptor TRPV1 [70]. Similarly, CBN has some antiproliferative effects in aggressive breast cancer cells [118], while CBC powerfully inhibits cell viability in both MDA-MB-231 and MCF-7 breast cancer cell lines, [70] and CBN has been revealed to have antiproliferative effects in aggressive breast cancer cells [127]. Among other terpenes, pinene shows anti-proliferative effects against MCF-7 breast cancer cells [34,128]. Several studies showed that limonene has anticancer effects on mammary carcinoma models causing regression and inhibiting subsequent tumor formation [129,130,131].

### 4.4. Colorectal Cancer

Normal and cancerous human colorectal tissues express both CB1 and CB2, indicating that cannabinoids have biological effects not only on colon but also on colorectal cancer. In this context, Aviello et al. showed that CBD exerts significant antiproliferative effects in Caco-2 and HCT116 colorectal carcinoma cell lines through induction of caspase 3 and apoptosis with an IC50 value reported as 7.5 ± 1.3 μM [132]. The molecular mechanisms underlying such effects are related to multiple pathways, such as mediated by CB(1)-, TRPV1, and PPARγ-antagonists sensitive manner [132]. Accordingly, Ligresti et al. reported that cannabinoid treatments decreased cell viability in undifferentiated Caco-2 cells via CB1 receptor. In comparison to the undifferentiated cells, CaCo-2 differentiated cells did not respond to cannabinoid treatments. It is interesting to note that the overall CB1 expression levels remained unchanged after differentiation [53,70,133].

In line with this study, it was demonstrated that HCT116 and DLD-1 colorectal cancer cells, treated with different concentrations of CBD, present elevated rate of apoptosis at treatment doses as low as 4 μM [134]; the selectivity of CBD actions have also been proved since no effects have been found in normal primary colorectal CCD-18Co cells and normal primary lung Beas2B cells. Regarding the mechanisms of death triggered by CBD, it has been shown that it induces Noxa-mediated apoptosis through the generation of ROS and excessive ER stress in both HCT116 and DLD-1 cells [134]. Jeong et al. also found that Noxa-activated apoptosis was dependent on excessive ER stress from ATF3 and ATF4 [134]. An additional pathway that, at least in part, contributes to CBD effects, is linked to autophagy-mediated death as well as to the arrest of cell cycle [126]. Kis et al. investigated the effects of CBD on the CT26 colon cancer cell line, showing that the beneficial effects of CBD are due to relevant antioxidant activity mediated by superoxide dismutase (SOD) and glutathione peroxidase (GPX) [1].

The possible use of phytocannabinoids in combination with different conventional therapies is gaining increased attention. Although oxaliplatin is an effective chemotherapeutic drug CRC treatment, patients often develop resistance to it; NOS3 is an essential molecular target for oxaliplatin resistance. The combinational treatment in vitro of CRC cells with oxaliplatin and CBD is able to decrease NOS3 phosphorylation, resulting in autophagy, and overproduction of ROS, thus overcoming oxaliplatin resistance [135].

It is relevant to notice that, both THC and CBD are able to restore the increase of the permeability and inflammation of intestinal cells, events typically occurring during the neoplastic process [136,137]; these THC and CBD effects suggest the regulation of inflammation as an additional mechanism for the anticancer effects. In line with this hypothesis, cytokines levels are significantly reduced by CBD treatment in in vitro models of colorectal cancer [138,139].

Regarding minor phytocannabinoids, CBG also stimulates apoptosis, ROS production, up-regulates C/EBP homologous protein (CHOP) mRNA, and inhibits cell proliferation in colorectal cancer cells [140]. Similarly, in Caco-2 cells, CBC can inhibit cell growth, but only at a concentration of 30 µM and CBDV reduces cell viability in a concentration-dependent manner, with an IC_50_ of 10 µM [141].

Myrcene extracts show significant cytotoxic effects in various tumors including breast carcinoma and colon adenocarcinoma [142] and other cell lines [143,144]. Similarly, in colon cancer cells, D-limonene suppresses cell viability through the induction of apoptosis via the suppression of the PI3K/Akt pathway [145].

## 5. *Cannabis sativa* L. in Cancer Clinical Trials

The role of *Cannabis sativa* in medicine is rapidly evolving. More than 30 countries worldwide have now legalized access to medical use of *Cannabis* [146,147]. A prospective observational study showed that many cancer-related symptoms improve significantly with *Cannabis* consumption. Similarly, another study, performed on over 3000 cancer patients, showed that cannabis use determines significant improvements in the control of common symptoms, including sleep problems (70.8%), fatigue (55.9%), anxiety and depression (74.1%), and nausea and vomiting (54.7%) [148].

Compared to THC, CBD-based preparations seem to be more promising, having diverse medicinal properties, such as anti-nausea, anti-emetic, anti-tumor, anti-inflammatory, antidepressant, anti-psychotic, and anti-anxiolytic effects [149]. To date, some information has been collected also in relation to the anticancer effects of CBD [92,150], as well as for the management of cancer pain, cancer-related anorexia and cachexia, and chemotherapy-induced nausea and vomiting (CINV) [151,152,153].

Clinical data are available about CBD use for the treatment of glioblastoma. A clinical trial analyzed the effect of CBD as a single agent against glioblastoma (clinical trial: NCT02255292) while another placebo-controlled phase II clinical trial analyzed the effect of the combination of THC and CBD as adjuvant in the chemotherapy (clinical trial: NCT01812603) [https://clinicaltrials.gov/ct2/show accessed on 30 April 2021]. Both trials report very promising effects in terms of cancer regression.

Similarly, a clinical trial, regarding 119 patients with different solid tumors (e.g., breast, prostate, and esophageal), was conducted over a four-year framed period: in 92% of the patients, a reduction in tumor size was obtained when CBD oil was administered [154].

Besides trials aiming at validating anti-cancer properties, the effects of CBD-based preparations on cancer pain patients are finding a wide interest [155,156,157,158,159]. A significant analgesic effect has been assessed in patients with malignant disease in 15 of 18 trials as compared to placebo [160]. Several data have demonstrated improved average pain score and an increased good quality of life [161,162,163]. Additionally, when using various THC dosages or synthetic analogs for cancer-related pain, an improved pain relief was found [164,165]. Even in cancer patients suffering from inadequate analgesia control with opioid therapy, a combination of THC and CBD can reduce pain score more than 30% from baseline [166,167], while the THC group showed a non-significant improvement [37]. However, the main limitation of THC remains sedation [168,169], while the long-term use of the THC/CBD spray is generally well-tolerated for as long as 2 years [170].

CB receptors, highly expressed in the neuronal tracts for emesis, have been chosen for treating CINV [171]. Results from several preclinical studies suggest that THC and CBD have anti-inflammatory, analgesic, anti-nausea, antiemetic, anti-psychotic, anti-ischemic, anxiolytic, and anti-epileptic impact [172,173,174]. In a small, controlled, randomized, “double-blind” experiment, oral THC reduces vomiting caused by chemotherapeutic agents [175].

Finally, the use of cannabinoids is linked to the increase of appetite and the gain of weight (ClinicalTrials.gov accessed on 14 March 2021, NCT02359123) [151]. However, cannabis extract and THC were tested in a Phase III clinical trial with no significant differences among patients with cancer-related anorexia-cachexia syndrome in terms of appetite [152]. More promising results have been later obtained that described a relevant increase in appetite [176]. Finally, in cachectic NSCLC patients, the use of a synthetic analog of THC, in a Phase II trial, significantly increases appetite and caloric intake [23]. No significant differences between THC/CBD and THC alone have not been observed [152].

## 6. Conclusions

**Figure 4:**
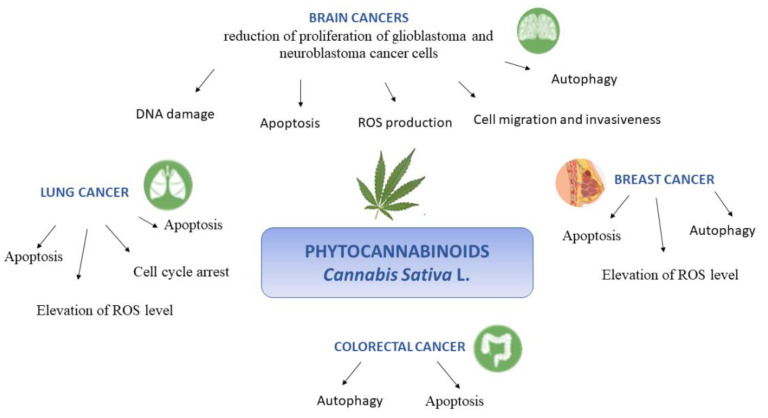
Schematic representation of the multiple molecular effects of phytocannabinoids from *Cannabis Sativa* L. against brain, lung, breast and colorectal cancer.

There is still urgent need of improving cancer treatment through the identification of a novel pharmacological drug. In this context, the phytocannabinoids from *Cannabis sativa* L. are receiving growing attention due to their promising therapeutic potential about the treatment of variety of cancers such as that affecting brain, breast, lung, and colon. Indeed, whether at present and undoubtedly, the cannabinoids are in use for the control of adverse reactions to conventional cancer treatments, an additional important direct role of these compounds in the development, progression, and metastasis of tumors is emerging. Furthermore, increasing the body of in vitro and in vivo evidence supports apoptosis, proliferation, and inflammation such as underlying mechanisms through which cannabinoids exert their anticancer effects (schematically reported in Figure 4). In addition, several evidence indicate that the activity of phytocannabinoids might be more effective in combinational therapies, encouraging to explore novel combinations and treatment schedules. However, the translation of cannabinoids use into clinical practice is still now in the initial phases.

Further studies will facilitate a better understanding of the effective application of cannabinoids in oncology.

## Abbreviations

THC	Δ9-tetrahydrocannabinol
CBD	cannabidiol
CBDA	cannabidiolic acid
THCA	tetrahydrocannabinol acid
CBN	cannabinol
AC	adenylyl cyclase
CB1 and CB2	cannabinoid receptor 1 and 2
cAMP	adenosine monophosphate
PKA	protein kinase A
MAPK	protein kinases
PI3K/AKT	phosphoinositide-3-kinase/protein kinase B
BBB	blood–brain barrier
OA	olivetolic acid
CBGAS	cannabigerolic acid synthase
CBGA	cannabigerolic acid
CBDAS	cannabidiolic acid synthase
Δ9-THCAS	Δ9-tetrahydrocannabinolic acid synthase
CBF-C5	cannabifuran
DCBF-C5	dehydrocannabifuran
CBCON-C5	cannabicoumaronone-C5
CBGVA	cannabigerovarinic acid
PUFA	polyunsaturated fatty acids
ALA	α-linolenic
GLA	γ-linolenic acid
SDA	stearidonic acid
ES	endocannabinoid system
eCBs	endogenous cannabinoid ligands
NAPE	N-acyl-phosphatidylethanolamine
NAPE-PLD	NAPE-specific phospholipase D
DAG	diacylglycerol
DAGL	DAG lipase
FAAH	fatty acid amide hydrolase
MAGL	monoacylglycerol lipase
TRPV1	transient receptor potential vanilloid 1
PPAR	peroxisome proliferator-activated receptor
TRP	transient receptor potential
TNF-𝛼	Tumor Necrosis Factor alpha
IL	Interleukin
NF-kB	nuclear factor kappa-light-chain-enhancer of activated B cells
COX-2	Cyclooxygenase 2
PGE2	Prostaglandin E2
NO	nitric oxide
TLR4	Toll like receptos 4
MMP	matrix metallopeptidase
VEGF	vascular endothelial growth factor
MDR	multidrug resistance
GBM	glioblastoma multiforme
GSC	glioma stem cell lines
ERK	extracellular signal-related kinase
PI3K	phosphoinositide3-kinase
p38MAPK	p38mitogen-activated protein kinase
ER	estrogen receptors
SOD	Superoxide dismutase
GPX	glutathione peroxidase
CINV	chemotherapy-induced nausea and vomiting
